# Chemogenomic library design strategies for precision oncology, applied to phenotypic profiling of glioblastoma patient cells

**DOI:** 10.1016/j.isci.2023.107209

**Published:** 2023-06-25

**Authors:** Paschalis Athanasiadis, Balaguru Ravikumar, Richard J.R. Elliott, John C. Dawson, Neil O. Carragher, Paul A. Clemons, Timothy Johanssen, Daniel Ebner, Tero Aittokallio

**Affiliations:** 1 Institute for Cancer Research, Department of Cancer Genetics, Oslo University Hospital, 0310 Oslo, Norway; 2 Centre for Biostatistics and Epidemiology (OCBE), Faculty of Medicine, University of Oslo, 0317 Oslo, Norway; 3 Institute for Molecular Medicine Finland (FIMM), HiLIFE, University of Helsinki, 20520 00290 Helsinki, Finland; 4 Cancer Research UK Scotland Centre, Institute of Genetics and Cancer, University of Edinburgh, Edinburgh EH4 2XR, UK; 5 Chemical Biology and Therapeutics Science Program, Broad Institute of Harvard and MIT, 415 Main Street, Cambridge, MA 02142, United States; 6 Target Discovery Institute, Nuffield Department of Medicine, University of Oxford, Oxford OX3 7FZ, UK

**Keywords:** Genetics, Cancer

## Abstract

Designing a targeted screening library of bioactive small molecules is a challenging task since most compounds modulate their effects through multiple protein targets with varying degrees of potency and selectivity. We implemented analytic procedures for designing anticancer compound libraries adjusted for library size, cellular activity, chemical diversity and availability, and target selectivity. The resulting compound collections cover a wide range of protein targets and biological pathways implicated in various cancers, making them widely applicable to precision oncology. We characterized the compound and target spaces of the virtual libraries, in comparison with a minimal screening library of 1,211 compounds for targeting 1,386 anticancer proteins. In a pilot screening study, we identified patient-specific vulnerabilities by imaging glioma stem cells from patients with glioblastoma (GBM), using a physical library of 789 compounds that cover 1,320 of the anticancer targets. The cell survival profiling revealed highly heterogeneous phenotypic responses across the patients and GBM subtypes.

## Highlights

• Systematic strategies for designing targeted anticancer small-molecule libraries
• Minimal screening library of 1,211 compounds for targeting 1,386 anticancer proteins
• Pilot screening study identified patient-specific vulnerabilities in glioblastoma
• Compound and target annotations and the pilot screening data made freely available

## Graphical abstract

**Figure:**
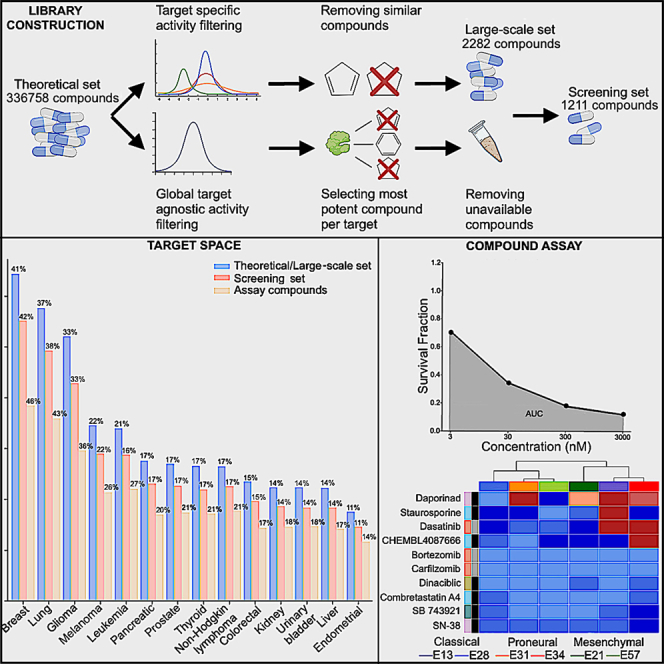

## Introduction

In the past ten years, there have been encouraging advances in the treatment and consequently, the survival rates, for many cancers. This progress has largely been driven by increased molecular understanding and classification of distinct cancer subtypes, the development of novel therapeutics focusing on targets associated with specific disease subtypes, and advances in both the type and range of therapeutic molecules available for the treatment of patients. When considering molecules that have been approved or advanced in clinical trials, there are successful examples of targeted chemotherapeutics,^1^ small interfering RNAs,^2^ monoclonal antibodies,^3^ microRNAs,^4^ and virotherapy,^5^ among other drug classes. These developments are very encouraging considering the complexity of human cancers and the difficulty in developing effective treatments for advanced disease. However, small-molecule chemotherapeutics still makeup the vast majority of approved drugs available to oncologists treating cancers.^6^^,^^7^^,^^8^ In the case of glioblastoma (GBM) brain tumors, small-molecule chemotherapeutics are currently the only approved treatment modalities beyond surgery and radiation^9^ and represent the most fertile ground for future innovation of new therapeutics to address the current challenges inherent in treating brain tumors. These challenges include (i) breaching the blood-brain barrier to effectively deliver therapeutics to the tumor, (ii) developing combinatorial treatments to systematically target redundant signaling pathways and tumor vulnerabilities inherent in brain tumors which typically exhibit wide intra- and inter-tumor heterogeneity, and (iii) selectively targeting GBM stem cells, which have been shown to be the main source for cancer recurrence in GBM.^10^

Traditional drug development often employs high-throughput drug screening of large collections of diverse small-molecule libraries against a nominated therapeutic target to identify chemical starting points (hit compounds) for further optimization. This process has been relatively successful at the industrial level, but it tends to become less successful at the academic level, due to the prohibitive infrastructural costs required to develop and prosecute large-scale screens,^11^ as well as the cost to develop hit compounds to viable leads and sequentially to an investigational drug candidate. Instead, many academic target discovery facilities have focused on developing more physiologically relevant phenotypic assays, which better recapitulate disease biology,^12^^,^^13^ and screening of smaller, more focused libraries of small compounds, such as collections developed and curated for drug repurposing,^14^^,^^15^^,^^16^ probes for target discovery,^6^^,^^17^^,^^18^ or pharmacologically active targeted compound sets for specific target classes.^7^^,^^19^^,^^20^ This focused approach has the advantage of providing a better understanding of the molecular basis of the disease, while simultaneously exploiting existing therapeutics and compounds with known safety profiles, along with probes that possess drug-like properties and compounds with known protein targets. In complex diseases of unmet therapeutic need, where target biology is still poorly understood or where disease heterogeneity indicates multiple target pathways that contribute to disease progression (as exemplified by GBM), phenotypic screening of target-annotated compound libraries in relevant patient-derived cell models may provide a valuable strategy for empirical identification of druggable targets or drug combinations. By circumventing major pitfalls, such as poor selectivity, cellular activity, and biological or target space diversity, these targeted libraries have great potential to accelerate the drug discovery process.

Here, we describe the construction of a comprehensive anticancer target-annotated compound library, designed to interrogate a wide range of potential cancer targets in phenotypic screening. The library design is approached as a multi-objective optimization (MOP) problem, where the aim is to maximize the cancer target coverage, while guaranteeing compounds’ cellular potency and selectivity, and minimize the number of compounds arrayed into the final screening library. To do this, we used two target-based design strategies. First, we searched for small molecules against the druggable cancer targets among approved and investigational compounds (AICs) identified from the literature, drug databases, and existing oncology collections. Second, to expand the target-annotated compound library, we surveyed several pan-cancer studies to identify anticancer compound-target pairs and then expanded the chemical space around those novel targets by identifying additional bioactive compound probes through database queries. Importantly, cancer-mutated proteins, nearest neighbors, and influencer targets were further investigated for potential small-compound interactors, which generated a large *in silico* probe set collection. Finally, we refined the probe set collection by applying several filters, with adjustable activity and similarity thresholds, and removed redundant structures and compounds which could not be readily sourced at the time of library curation, to yield a sufficiently diverse, focused, and target-annotated compound library for phenotypic screening purposes, named the Comprehensive anti-Cancer small-Compound Library, or **C**^**3**^**L**. In the pilot application of C^3^L to cell survival profiling of patient-derived GBM stem cell models, we discovered widely heterogeneous patient-specific vulnerabilities and target pathway activities. All the compound libraries and their target and compound annotations, as well as the pilot screening data, are freely available as data spreadsheets and through an interactive web platform (www.c3lexplorer.com).

## Results

### Identifying and curating small-molecule inhibitors of cancer-associated targets

**Figure 1:**
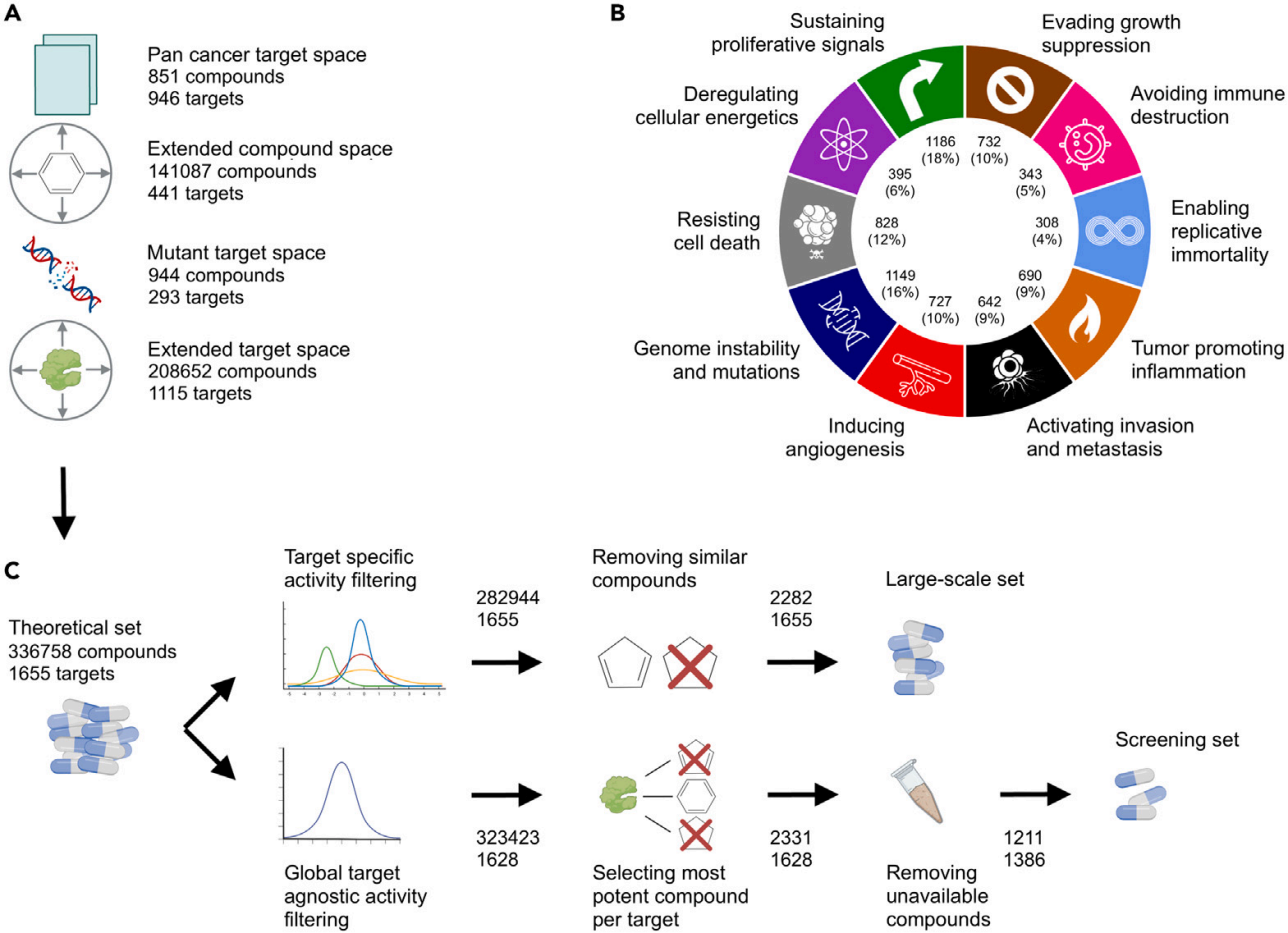
Target-based pipeline for designing of the three experimental probe compound (EPC) sets (Theoretical, Large-scale, and Screening set) (A) Four target-based compound collections were defined: (i) the pan-cancer collection includes compounds and their annotated targets from various pan-cancer studies; (ii) the extended compound space consists of additional compounds that have off-target activity against the annotated targets based on bioactivity data from public drug/target repositories such as ChEMBL,^25^ Drug Target Commons (DTC),^26^ and DrugBank;^27^ (iii) the mutant target space compounds have activity against the mutant variants of the annotated targets extracted from the COSMIC database;^28^ (iv) the extended target space includes compounds with activity against cancer-related targets established through a nearest neighbor approach.^29^ (B) The targets of the compounds in the theoretical set cover all the hallmarks of cancer. *Note*: each target protein may belong to multiple hallmarks explaining why they do not add up to 1,655. (C) The filtering criteria for designing the large-scale and physical screening sets. See STAR Methods for the step-by-step procedures for the construction of the various probe set collections.

Our first design objective was to define a comprehensive list of protein targets associated with the development and progression of cancers to form the basis of the anticancer small-molecule library. We first defined a list of proteins known to be implicated in cancers using The Human Protein Atlas^21^ and nominal targets of pan-cancer studies from the PharmacoDB,^22^ leading to the target space of 946 oncoproteins (Figure 1A; see STAR Methods for details). We then expanded the target space by using additional pan-cancer studies linked back to cancer-related targets to define the full set of 1,655 proteins or other cancer-associated gene products. Our target space was designed to span a wide range of protein families, cellular functions, and cancer phenotypes, and it covers all the categories of “hallmarks of cancer”^23^^,^^24^ (Figure 1B).

After defining the comprehensive list of cancer-associated targets, our next objective was to identify and curate a small-molecule collection of compounds targeting these proteins. Since the compounds ranged from investigational and experimental probe compounds (EPCs) to approved drugs, we took a systematic approach to defining each source, through sorting the compounds targeting the cancer-associated targets, ranking the compounds for activity, diversity, and availability, and finally, producing a sortable and searchable database of the screening library along with its target and other annotations. The screening library construction started from >300,000 small molecules and ended up with 1,211 optimized for physical library size, cellular activity, chemical diversity, and target selectivity, leading to 150-fold decrease in compound space, yet still covering 84% of the cancer-associated targets.

### Target-based approach: EPC collection

Using the target-based design, we extracted compound-target interactions manually from public databases, leading to chemical probes and investigational compounds in three nested subsets of gradually decreasing sizes; (i) the **theoretical set** is an *in silico* set curated from established target-compound pairs covering the expanded target space of 1,655 cancer-associated proteins, (ii) the **large-scale set** is a broader screening collection of filtered compounds covering the same target space as theoretical set, and (iii) the **screening set** is the final set of most potent probes arrayed into the physical library. The screening set is the smallest subset due to the limitations in compound commercial availability for screening purposes. Figure 1C shows a schematic diagram of the construction of these three target-based compound sets, with their compound and target numbers.

The **theoretical set** contains 336,758 unique compounds from four probe sets for pan-cancer target space, pan-cancer collection with extended compound space, mutant target space, and mutant target collection with extended target space. The **large-scale set** contains a subset of 2,288 compounds from the theoretical set, filtered to reduce the number of molecules in the library while still covering the same target space, using both the activity and similarity filtering procedures with pre-defined cutoff values (see Figure S4 for the filtering parameters used in this study, but the cutoff parameters are freely adjustable for other studies and library constructions). The large-scale set was based on both the on- and off-target profiles of the 2,282 small-molecule compounds, which could be used in larger-scale screenings campaigns in academic or industrial projects (Table S1).

The **screening set** contains a smaller number (1,211) of compounds that are more easily purchasable and used for screening applications, thus making the probe set suitable for routine exploration of oncology-associated biological target space in complex phenotypic assays and identification of potential candidates for further drug development. This probe subset was obtained by subjecting the theoretical set to three filtering procedures (see Figure 1C). These filtering procedures involved (i) global target-agnostic activity filtering to remove 13,335 non-active probes, (ii) selecting the most potent compounds for each target to reduce the library to 2,331 compounds, and (iii) filtering by the availability of the compounds, which reduced the library size by 52%, while the target coverage remained at 86% and the target activity distributions were relatively unchanged (p > 0.05; Kolmogorov-Smirnov [K-S] test; Figure S1).

### Drug-based approach: AIC collection

**Figure 2:**
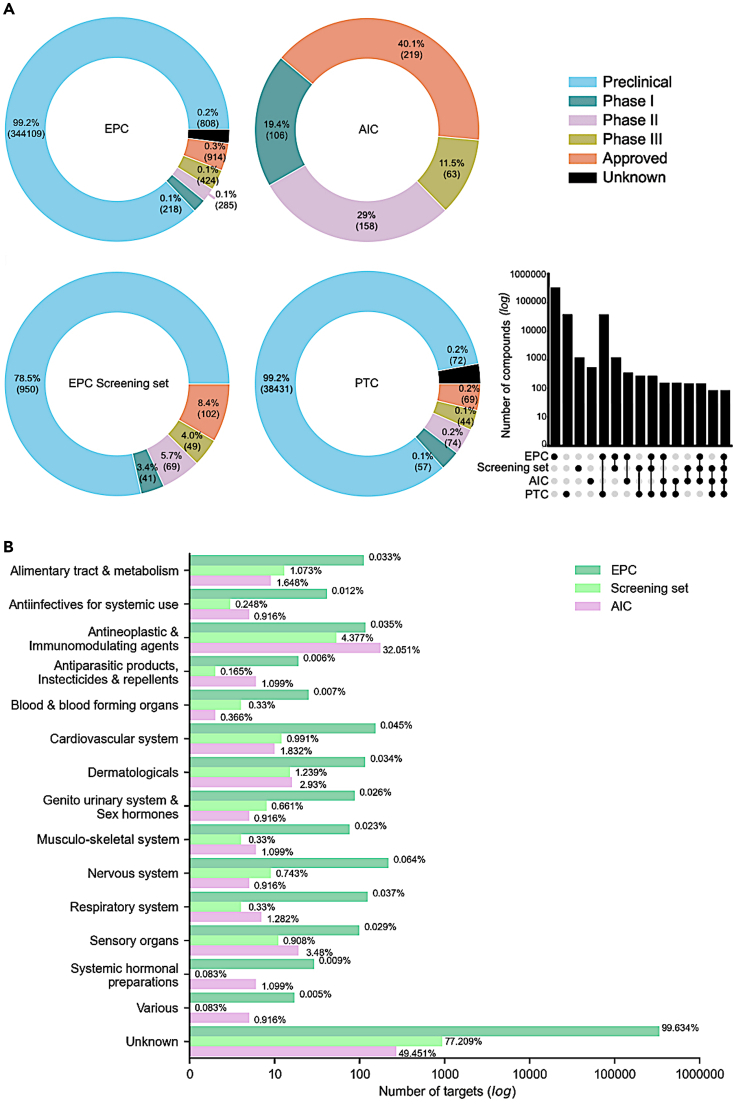
Compound spaces of the target-based and compound-based collections (A) Clinical development phases of the compound collections: target-based approach - experimental probe compound (EPC) collection, compound-based approach - approved and investigational compound (AIC) collection, and CRISPR9-based approach - priority target compound (PTC) collection (compounds targeting the 628 priority targets from the DepMap project). The clinical development phase was extracted from ChEMBL.^25^ Numbers in parentheses indicate the number of compounds in each category. The upset diagram shows the size of the intersections between the compound sets. The sets that are part of an intersection are filled in, and when only a single set is filled in, that indicates the number of compounds in that set. (B) The distribution of compounds in the collections in terms of the anatomical therapeutic chemical (ATC) classes, which distinguishes the organ or system on which the compounds act and their therapeutic, pharmacological, and chemical properties.

As expected from the target-based approach (i.e., identifying established potent small molecules for respective targets), EPC collections included mostly compounds that are currently in preclinical stages (Figure 2A). We next compared EPC collection with an additional set of small molecules that are currently approved for clinical use, also including anticancer compounds in various clinical development stages that might be candidates for drug repurposing applications. This compound-based design strategy led to a complementary AIC collection, named AIC collection, manually curated from several public compound sources and clinical trials. The AIC collection was further subjected to removal of duplicate molecules and similarity searches using the extended connectivity fingerprint (ECFP4/6) and molecular ACCess system (MACCS) fingerprints, where Dice similarity for ECFP4/6 and Tanimoto similarity for MACCS keys with cutoff of ≥ 0.99 were used to identify and remove structurally highly similar compounds (e.g., doxorubicin and epirubicin).

The AIC collection consisted of 546 unique compounds, out of which 74 (14%) are labeled as standard chemotherapeutics (Figure S2). Most of the molecularly targeted compounds in AIC collections are kinase inhibitors (50%), but the collection also includes various other drug classes, such as immunomodulatory agents (3.7%) and metabolic modifiers (2.9%). As expected, the AIC compounds contain a large number of approved drugs (40.1%), and the compounds in this collection only partly overlapped with those of the EPC collections (64% with EPC and 27% with screening set; Upset plot in Figure 2A). However, even if the total number of compounds is rather different in the collections, the AIC and EPC collections cover the same anatomical and therapeutic classes (ATCs), and the proportions of ATCs in the screening set were somewhere in between the AIC and EPC collections (Figure 2B).

To further compare the target-based EPC and compound-based AIC collections to a functional genomics-based approach to construct an anticancer compound library, we identified potent inhibitors of the 628 priority targets extracted from the CRISPR-Cas9 screens of the DepMap project,^30^ using a similar procedure to the one implemented for the EPC collection (see STAR Methods). The priority target compound (PTC) collection of 38,747 compounds included mainly preclinical compounds (Figure 2A), with a large number of compounds overlapping with the EPC collection (99%). The four compound collections had a core set of 85 common compounds shared by all the sets (Figure 2A, upset plot). Strikingly, PTC collection covers only 113 out of the 628 priority targets (18%), indicating that most of the loss-of-function screen targets are not currently druggable. The EPC screening set has more approved compounds (102, 8.4%), compared to the PTC collection (69, 0.2%).

### Characterization of the compound and target spaces of the probe collections

**Figure 3:**
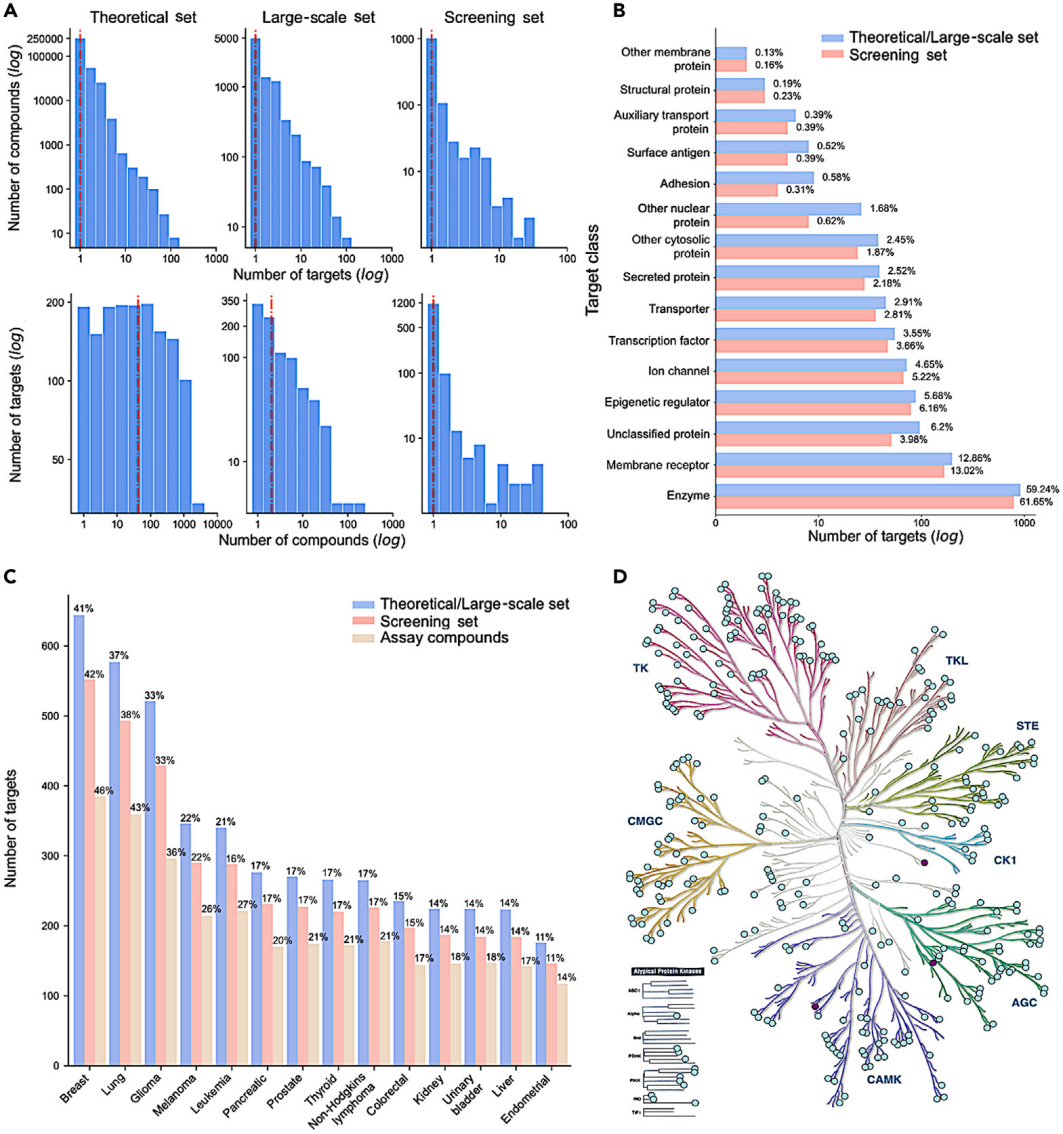
Target spaces of the compound collections (A) The number of targets per compound reported in drug databases (upper panel) and the number of compounds per target (lower panel) in the three compound collections. The dashed line indicates the median. The x axis and y axis are log_10_-scaled in each panel, while the numbers present the non-logged counts (note the different scales between the panels). The counts are based on the target activity threshold of ≤1000nM (see Figure S7 for the other activity thresholds). (B) The distribution of target classes of compounds in the screening set and theoretical/large-scale sets (that contain the same set of targets). The numbers next to the bars indicate the percentage of targets in the two collections of different sizes. The target classification of proteins was extracted from ChEMBL.^25^ (C) The number of targets associated with cancer types in the theoretical/large-scale, screening set and in the physical compound set that was tested in the patient-derived GBM stem cells in an imaging-based assay. The numbers above the bars indicate the percentage of targets in the two collections of different sizes. The disease associations were extracted from OpenTargets,^31^ with overall association score >0.5 (https://www.opentargets.org/). (D) Kinase families covered by the targets in the probe sets. The kinases colored in purple indicate the targets present only in the theoretical/large-scale sets, but not in the screening set, while the light-green targets are covered by all the three collections. KinMap^32^ web-tool was used for the creation of the illustration, reproduced courtesy of Cell Signaling Technology, Inc. (www.cellsignal.com).

After comparing the EPC collections to the approved/investigational and priority target collections, we next analyzed the compound and target spaces of the three EPC collections, designed using the target-based procedures and selected parameter values for filtering. The target distributions of the three probe collections remained relatively similar, whereas the screening set showed reduced numbers of multi-target compounds by its design (Figure 3A, upper panel). However, the median number of targets per compound was one for each probe collection, indicating that the sets include relatively selective compounds. For most of the targets, the screening set contains only a single potent compound, again per its design principles, whereas in the theoretical and large-scale sets the median number of compounds per target was 42 and 2, respectively (Figure 3A, lower panel).

The target class distribution of the screening set is similar to that of the larger probe collections of the theoretical and large-scale sets (K-S test, p = 0.19; Figure 3B). This indicates that the compound filtering did not miss any target class. As expected, targeted inhibitors of kinases and other enzymes are the most frequent, but there are also other well-covered target classes, such as membrane receptors, epigenetic regulators, and ion channels (Figure 3B). When zooming into kinases, the screening set covers all the major kinase families, and only 3 of the kinase targets present in the theoretical/large-scale sets are missed in the smaller screening set (Figure 3D). Importantly, the compound sets cover similar proportions of targets implicated in multiple types of cancers (K-S test, p = 1.0), with breast and lung cancers having the largest numbers of targeted compounds followed by glioma (Figure 3C).

The target distribution across cancer types remained very similar in the constructed physical library, which included 325 of the screening set compounds, and was used here in the pilot screening study on patient-derived GBM stem cells (assay compounds, Figure 3C), described in the next section. At the time of the curation, 325 compounds from the optimized library were sourced first from collaborators and then from multiple commercial providers. The remaining 886 compounds could not be sourced as they were not available from any vendor, were prohibitively expensive, or the lead time for acquisition of the compound was excessive. In these cases, we selected the most potent and selective inhibitor instead, yielding a completed C^3^L of 789 small molecules, covering 1,320 protein targets and 79% of the cancer-associated targets.

### Identification of compound responses across GBM subtypes in primary patient cells

**Figure 4:**
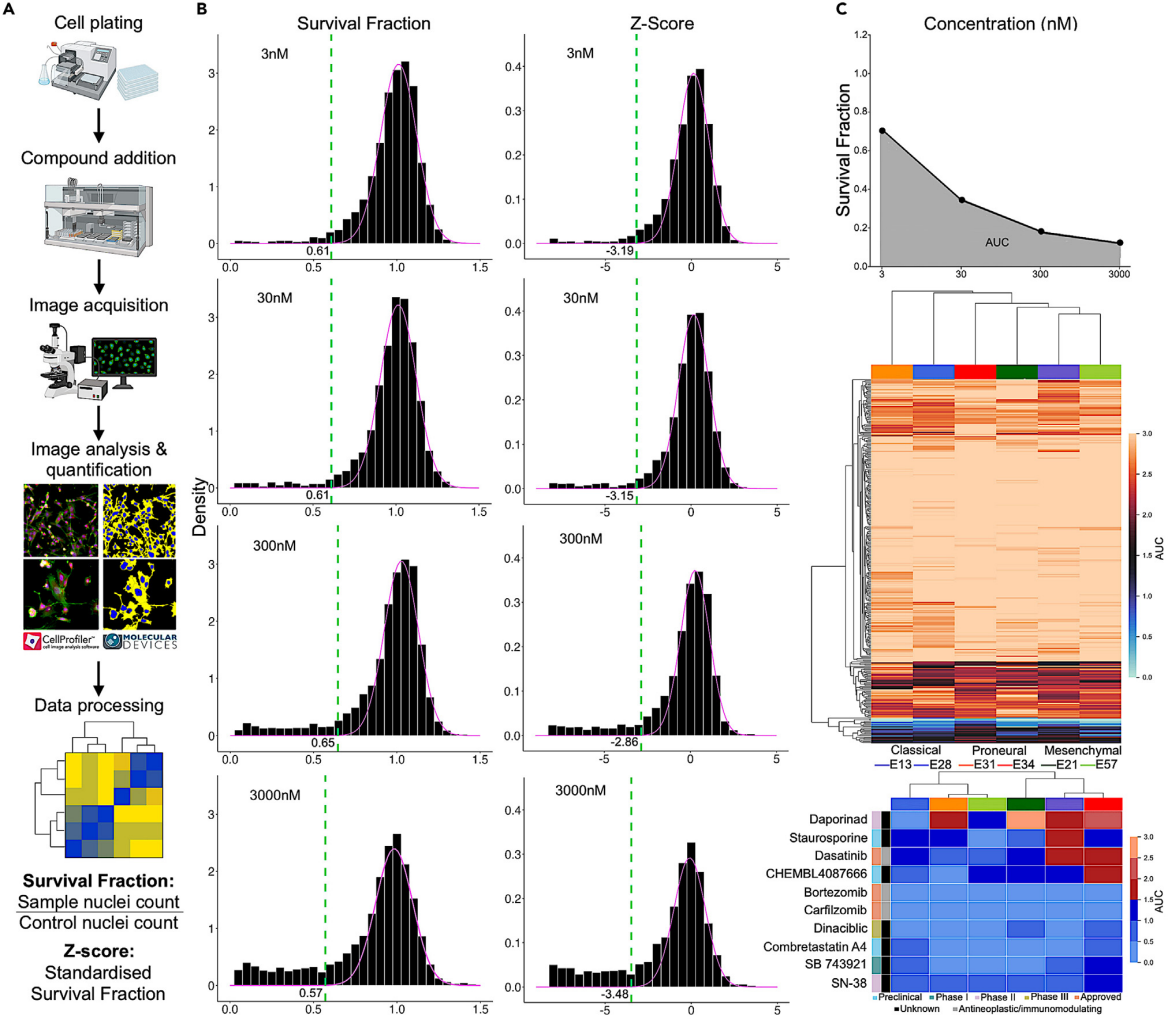
Cell survival profiling and data analysis (A) The imaging-based workflow to quantify patient-specific phenotypic features (see STAR Methods for details). (B) Examples of survival fraction distributions across 4 concentration levels of two patient-derived cell lines from two GBM subtypes: E34 (proneural) and E57 (mesenchymal). Lower values of survival fraction indicate higher compound activity. The green dashed line indicates the data-driven activity threshold for classifying a compound as a strong hit at the particular concentration level, when using the activity cutoff of 0.01 percentile of the background distribution. A total of 10 compounds with responses under the activity thresholds across all the 4 concentrations were identified as strong hits and were selected for further analysis. (C) Calculation of the area under the dose-response curve (AUC). Lower AUC values indicate higher activity of the compound. Two heatmaps of the AUC for all the 325 compounds from the screening set (upper heatmap) and for the 10 strong hits (lower heatmap, corresponding to the lowermost cluster of the full dendrogram) across 6 glioblastoma patient primary samples. The sample colors indicate the GBM subtypes, and the compound colors mark the clinical phase and ATC classes.

In a pilot screening application to identify therapeutic vulnerabilities in GBM, we carried out phenotypic profiling of patient-derived stem cell lines from 6 GBM patients covering the three GBM subtypes:^33^ classical (n = 2), proneural (n = 2), and mesenchymal (n = 2). The imaging-based compound testing assay quantifies the phenotypic effects of each compound by tracking cell proliferation or survival in response to the 789 compounds in the C^3^L physical library, each tested with 4 concentrations (see STAR Methods for details). These compounds and their 1,320 anticancer targets cover 27% and 79% of the compound and target spaces of the screening set, respectively. In this pilot screening study, we used a single phenotypic readout for further analysis: the survival fraction and its *Z* score, calculated based on nuclei counts in treated, untreated, and negative control DMSO samples (Figure 4A). The lower these readouts are, the more effective is the compound in the sense that fewer cancer cells survived the tested treatment.

We developed a data-driven method to define an activity threshold separately for each patient and dose, based on mixture modeling and parameter estimation (Figure 4B, see STAR Methods for details). Using a cutoff of 0.01 percentile of the background distribution, we identified as strong hits those compounds with responses under the data-driven activity thresholds across all the 4 concentrations. When using either the survival fraction or *Z* score at 0.01 percentile, we identified 10 strong response compounds from the screening set that cover multiple target classes. The benefit of such a data-driven threshold is that it provides an objective way to select patient-specific compounds that show strong activity at various concentration levels since it is estimated from the distribution of the measurements. Notably, the activity thresholds varied between the patients and dose levels, supporting the need for data-driven approach (Figure 4B).

To summarize the activity of a compound across the 4 tested concentrations, we calculated the area under the dose-response curve (AUC) for each compound-patient pair using the survival fraction as the response readout (Figure 4C, upper panel). The patient-specific response profiles showed a relatively large variability between the cell lines, even from the same GBM subtype, and the compound response profiles did not cluster according to the GBM subtypes (Figure 4C, middle panel). This supports the notion that GBM is a heterogeneous disease, also in terms of phenotypic responses to compound treatment, hence requiring patient-specific treatment approaches. The identified 10 strong hits included 3 antineoplastic and immunomodulating agents, such as approved multiple myeloma treatments bortezomib and carfilzomib, which showed an overall high potency in all the 6 GBM patient-derived cell models (Figure 4C, bottom panel).

### Screening hits identify patient-specific vulnerabilities and target pathway activities in GBM

**Figure 5:**
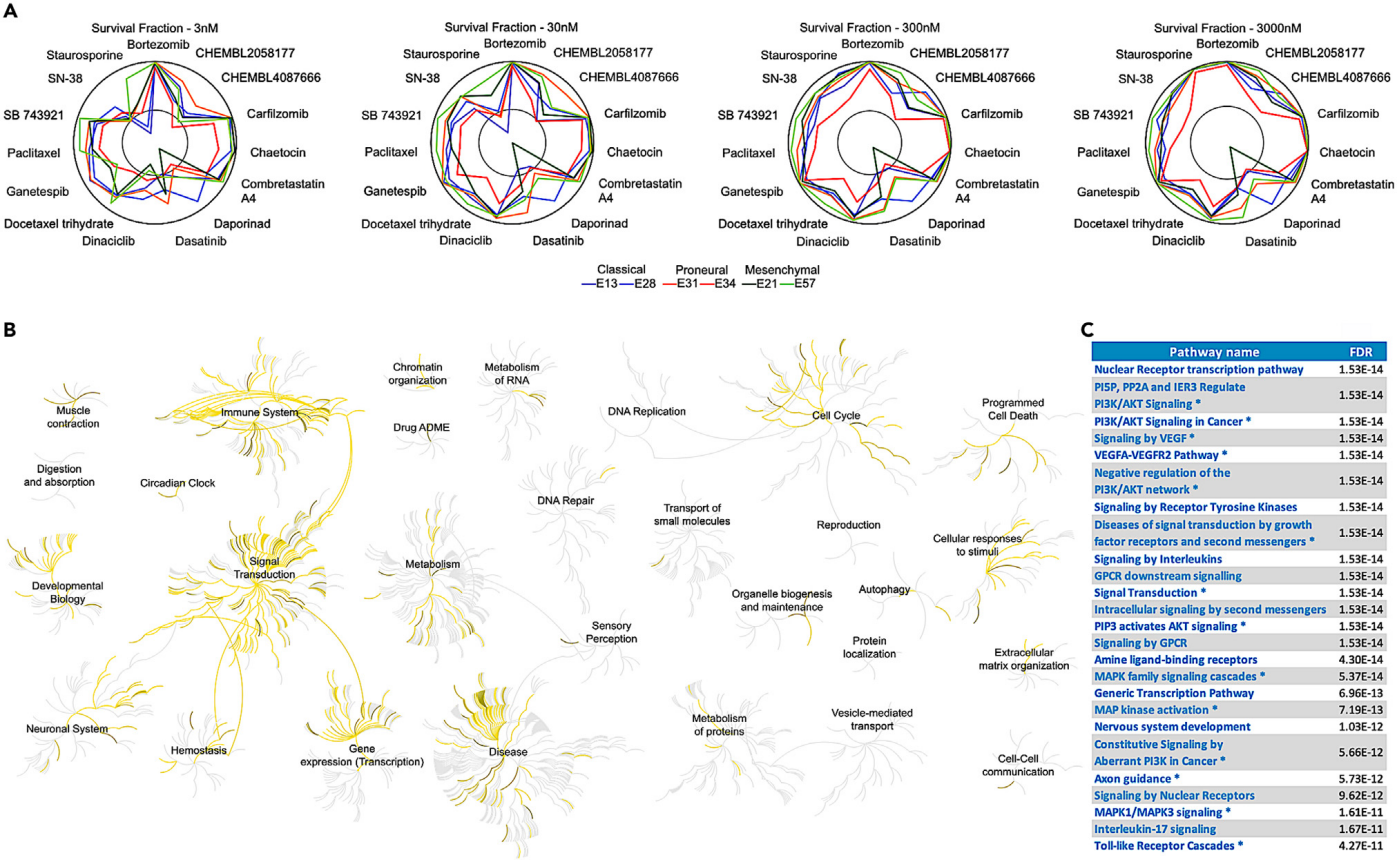
Analysis of the top hits from the patient screening (A) Radial plots of the 10 strong hit compounds and their survival fractions across the 4 concentration levels and 6 glioblastoma patient samples. Responses closer to the outer circle indicate lower survival fraction and therefore higher response. The inner gray circle marks the data-driven activity threshold of 0.01 percentile for the particular concentration level. (B) Reactome target pathway activities related to the 10 strong hit compounds. (C) The top 25 Reactome target pathways ranked by their false discovery rate (FDR). ∗Pathway enrichment also identified based on the KEGG pathways.

When investigating the 10 strong hit compounds across the concentration levels and patient samples, one can see an expected dose-response relationships, where higher concentrations lead to increased responses of the compounds (Figure 5A). However, the compounds have differing activity profiles both within and between the GBM subtypes. For instance, dinaciclib, an investigational compound that inhibits cyclin-dependent kinases (CDKs), showed high activity in multiple patients already at lower concentrations (30nM), except for one proneural subtype patient. The broad efficacy of dinaciclib was shown recently also in 2D and 3D GBM models and in long-term culture experiments.^34^ In contrast, another broadly active kinase inhibitor dasatinib induced more mixed responses across the GBM subtypes, in contrast to earlier studies that have reported that patients with the mesenchymal subtype show more sensitivity to dasatinib compared to proneural and classical subtypes.^35^ Notably, nicotinamide phosphoribosyltransferase (NMPRTase) inhibitor daporinad demonstrated highly selective response in a single patient of classical subtype, while the other patients had much lower responses to daporinad at all concentrations (Figure 5A).

Pathway analyses among the protein targets of strong hit compounds demonstrated that these compounds modulate multiple cellular pathways, including immune system, signal transduction, neuronal system, cell cycle, and cellular responses to stimuli (Figure 5B). There are also less-pronounced pathway activities that originate from more selective responses; for instance, daporinad triggers autophagy. More detailed pathway enrichment results from both Reactome and Kyoto Encyclopedia of Genes and Genomes (KEGG) highlight PI3K/Akt/mitogen-activated protein kinase (MAPK) signaling as one of the top-enriched pathways (Figure 5C), which is important in GBM as ∼40% of GBM patients have phosphatase and TENsin homolog (PTEN) alterations (in our panel, 4 out of 6 [66.6%] of the patient-derived cells had PTEN alteration), as well as frequent neurofibromatosis type 1 (NF1) loss (E57 cells) or alterations to the epidermal growth factor receptor (EGFR) receptor (amplification in 4 out of 6 patient-derived cells; missense mutation in the E31 cells). To enable others to explore these data and freely set other data-driven thresholds for the compound selection, we have implemented a web application (www.c3lexplorer.com) that allows the users to interactively explore the signaling and other pathways targeted by the selected compounds for additional analyses and hypothesis generation.

## Discussion

Phenotypic screening provides a systematic approach to identify novel small molecules as promising future therapeutics, especially when focusing on realistic disease models and relevant target landscapes.^36^ In this work, we developed various analytical procedures for the construction of targeted anticancer compound libraries, designed to interrogate a wide range of potential cancer-related targets for phenotypic screening. We started the systematic library construction from more than 300,000 molecules, by compiling comprehensive chemical, activity, and target information, and ended up with a physical screening set of 1,211 molecules with pre-defined cellular activity, biological and chemical diversity, target selectivity, and compound availability. This reduced the compound space by 150-fold, whereas the remaining compounds in the screening set still covered 84% of the target space of 1,655 cancer-related proteins. The remaining 16% represent targets that need further development due to a lack of either enough specific and potent chemical probes (2%) or compound availability for sale (14% at the time of library curation).

We applied the C^3^L design principles to imaging-based phenotypic profiling of glioma stem cells from GBM patients, using a pilot library of 789 compounds, and identified patient-specific compound responses and target pathway activities. We anticipate these systematic library-design principles and the resulting broadly annotated small-molecule libraries will prove useful for the community in various phenotypic screening experiments in GBM and other cancers. We have made the compound libraries and their annotations available in the form of open-access data sheets (https://github.com/PaschalisAthan/C3L) and implemented a web platform that enables others to interactively explore the screening library and corresponding compound-target interaction networks, as well as screening data and pathway activities using user-defined activity thresholds and other filtering criteria (www.c3lexplorer.com). This enables the community to access these data and make further analyses, e.g., drug class-specific responses or for identifying combinatorial treatments to target multiple pathway redundancies and patient-specific intra-tumor vulnerabilities that are urgently needed for treating patients with GBM.^37^

In the field of precision oncology, there have been several efforts to systematically catalog all of the genes implicated in cancer,^38^^,^^39^ and several in-house, commercial, and governmental libraries of AICs targeting many of the most heavily investigated proteins are readily available.^14^^,^^15^^,^^40^^,^^41^^,^^41^^,^^41^^,^^42^^,^^43^^,^^44^^,^^45^^,^^46^ However, even though these commercial or in-house libraries have been well-utilized in many anticancer screens and drug repurposing studies,^16^^,^^17^^,^^47^^,^^48^ there is a need for a comprehensive, target-annotated, anticancer library optimized for selectivity and diversity against all known oncology targets. There are well-annotated libraries for specific classes or applications, such as for kinase inhibitors,^49^ metabolic modifiers,^20^ and drug repurposing,^50^ but what is lacking is a comprehensive and high-quality probe library that provides a starting point for various anticancer screening applications. Despite the emergence of new treatment modalities, such as monoclonal antibodies, molecularly targeted small molecules are, and will most likely remain, the most prolific anticancer therapeutics for the foreseeable future that are also applicable in advanced stages of the disease, for instance, after tumor has progressed toward metastatic disease and when the cancer has become chemo- or radiotherapy resistant.^6^^,^^7^^,^^8^

The development and implementation of well-curated and comprehensive screening libraries are expected to facilitate the identification of novel anticancer drug targets, drug combinations, synthetic-lethal interactions, and therapeutic targets to overcome cancer resistance that goes beyond the recurrently mutated genes.^51^ To enable cross-comparisons, we have carefully annotated our libraries with multiple compound IDs for molecules and target annotations; these include unique ChEMBL ID that makes it easy to extract additional information of the compounds, compound generic name of the active ingredient, chemical abstracts service (CAS) registry number for sourcing the compounds from potential vendors, and simplified molecular input line entry system (SMILES) for describing the structure of chemical species for further chemical analyses. We have also carefully annotated the active on/off-target space of the compounds using multiple databases, such as ChEMBL, DrugBank, and DrugTargetCommons, using both gene accession and gene symbol of the target proteins. Such annotations make it easy for others to further characterize the chemical and target spaces of the compound libraries, as well as compare with the existing and emerging libraries to allow for comparative analyses of their shared and unique chemical and target spaces for future library developments and screening applications.

We formulated the compound library construction as an MOP problem, where the aim is to simultaneously maximize the anticancer target diversity and compound potency (or selectivity), while minimizing the number and cost of the compounds in the physical screening set. This is important because a targeted library enables a more thorough evaluation of the anticancer potential in advanced, physiologically relevant cancer models, such as multiple genetically distinct patient-derived cell panels. With such a focused yet diverse-enough library, comprehensive screening paradigms including drug combinations, sequencing, and scheduling can be more easily incorporated into a primary phenotypic screening pipeline, achievable by many academic screening facilities.^52^ In the present work, we used fast and heuristic procedures to select the compounds with a pre-defined potency against the selected targets that presented with sufficient structural dissimilarity, as implemented in several filtering procedures. Even though our results show that the current heuristic approach leads to the desired results from the compound screening point of view, other approximated or exact solutions to the MOP problem could further decrease the size of the screening set, while guaranteeing sufficient potency and selectivity of the compounds against the disease-related and other targets.^53^

Designing an optimal compound library of small molecules is challenged by the compound promiscuity; that is, many compounds may modulate their effects through multiple protein targets with various degrees of potency in a given context. For instance, kinase inhibitors are notorious for their target promiscuity and wide polypharmacological effects across various target classes beyond kinase families.^54^ The wider target selectivity of many compounds remains still uncharted,^54^^,^^55^ and therefore the phenotype-driving targets of many compounds are currently still unknown in many cellular contexts or disease applications. Compared to other compound libraries that focus more on compound selectivity, C^3^L was designed based on dual objectives: (i) to identify compounds that are effective against GBM or other cancers (either individually or in combination) and (ii) C^3^L function as a target-identifying or hypothesis-generating library for follow-up studies. In the first application, target specificity is not as important as compound efficacy. In the second application, specificity is more important but not a necessary condition for hypothesis generation. Systematic study of cross-reactivity of the compounds will be needed to further investigate the target selectivity of the molecules in the current screening set across various target classes and cell contexts, beyond the rather limited data for target activity currently available in public databases.

### Limitations of the study

We highlight below some potential caveats of the present work and how the library design could be further improved in the future studies. In addition to the potency and selectivity, chemical similarity of the compounds is another important feature of library design, especially if the aim is to have a collection of not too similar small molecules that target the cancer-related proteins of interest. In addition, further phenotypic and clinical characterization of the compounds, as implemented in Drug Repurposing Hub^50^ and ChemicalChecker,^56^ could aid several drug discovery tasks, including target identification, mechanism of action (MoA) classification, and library characterization. Integrated analysis of chemical, molecular target, cell-based profiling, and clinical information could also be useful to provide a relevance ranking of the targets and compounds in a library, once the disease application is defined, hence adding one further dimension to the MOP problem. Additional filtering steps to aid the eventual screening applications include, for instance, removal of interference compounds, finding on-target compounds with diverse scaffold profiles, and exclusion of compounds with known adverse off-target activities. The relatively small size of the physical screening library used in the pilot screen may miss some relevant compounds and targets in GBM. To investigate and improve the relevance of the hits, follow-up phenotypic screens with full morphological cell painting profiling are warranted.

The commercial availability of the compounds was a limiting factor that reduced the physical library size significantly. Inclusion of synthesizable compounds from the theoretical set can further increase the diverse and coverage of the screening set in the future versions. Even though screening of compounds with overlapping primary targets increases the cost, having multiple compounds with overlapping primary targets may become beneficial in specific applications, e.g., for validating the screening hits from high-throughput screens, target deconvolution of the phenotypic responses, or for discovering target activity differences when screening at multiple concentrations. The future applications in phenotypic screening, either in established cell lines or patient-derived cell models, will define the usefulness of any compound collections and libraries and the cost-benefit trade-off of compound libraries with various sizes and target overlaps. We expect that the herein presented comprehensive libraries with curated target information and the pilot screening data in patient-derived cells will provide new opportunities for many exciting drug discovery applications in GBM and other cancers, including novel target identifications, target deconvolution, drug repositioning, and drug combination prediction and testing.^57^ Follow-up studies of the screening hits may lead to the identification of chemical starting points, new target-directed drug discovery programs, or approval of new therapeutics for GBM and other cancers.

## Supporting citations

^63^ ^,^ ^64^

## STAR★Methods

### Key resources table

**Table:** 

REAGENT or RESOURCE	SOURCE	IDENTIFIER
**Biological samples**		

Patient derived Glioma stem cells	Glioma Cellular Genetics Resource	http://gcgr.org.uk

**Chemicals, peptides, and recombinant proteins**		

Cultrex 3D matrix Laminin	R&D Systems rndsystems.com	3446-005-01
Hoechst-33342	MolProbes	H1399

**Software and algorithms**		

TIBCO Spotfire Analyst	Spotfire.tibco.com	N/A
Imagexpress IXM (with built in software/image analysis modules)	Molecular Devices, moleculardevices.com	N/A
PostgreSQL	Postgresql global development group	https://www.postgresql.org/support/
Python 3.7	Van Rossum G and Drake F.L. Jr	https://www.python.org/
RDKit	Landrum G.	http://www.rdkit.org.
ShinyGO	Ge, S.X., Jung, D., and Yao, R.	http://bioinformatics.sdstate.edu/go/

**Other**		

Microplates 384w uCLEAR Black, F-Bottom, TC, Sterile	Greiner Bio-One. gbo.com	781091
Integra ViaFill Mulitfunctional Reagent Dispenser	Integra Biosciences	N/A
PubChem	Kim, S., Chen, J., Cheng, T., Gindulyte, A., He, J., He, S., Li, Q., Shoemaker, B.A., Thiessen, P.A., Yu, B., et al.	https://pubchem.ncbi.nlm.nih.gov/
ChEMBL	Gaulton, A., Hersey, A., Nowotka, M., Bento, A.P., Chambers, J., Mendez, D., Mutowo, P., Atkinson, F., Bellis, L.J., Cibrián-Uhalte, E., et al.	https://www.ebi.ac.uk/chembl/
SelleckChem	Selleck	https://www.selleckchem.com/screening/anti-cancer-compound-library.html
PharmacoDB	Smirnov, P., Kofia, V., Maru, A., Freeman, M., Ho, C., El-Hachem, N., Adam, G.-A., Ba-Alawi, W., Safikhani, Z., and Haibe-Kains, B.	https://pharmacodb.ca/
The Human Protein Atlas	Uhlén, M., Fagerberg, L., Hallström, B.M., Lindskog, C., Oksvold, P., Mardinoglu, A., Sivertsson, Å., Kampf, C., Sjöstedt, E., Asplund, A., et al.	https://www.proteinatlas.org/
DrugTargetCommons	Tang, J., Tanoli, Z.-U.-R., Ravikumar, B., Alam, Z., Rebane, A., Vähä-Koskela, M., Peddinti, G., van Adrichem, A.J., Wakkinen, J., Jaiswal, A., et al.	http://drugtargetcommons.fimm.fi/
DrugBank	Wishart, D.S., Feunang, Y.D., Guo, A.C., Lo, E.J., Marcu, A., Grant, J.R., Sajed, T., Johnson, D., Li, C., Sayeeda, Z., et al.	https://go.drugbank.com/
COSMIC	Forbes, S.A., Bhamra, G., Bamford, S., Dawson, E., Kok, C., Clements, J., Menzies, A., Teague, J.W., Futreal, P.A., and Stratton, M.R.	https://cancer.sanger.ac.uk/cosmic
SignaLink v2	Fazekas, D., Koltai, M., Türei, D., Módos, D., Pálfy, M., Dúl, Z., Zsákai, L., Szalay-Bekő, M., Lenti, K., Farkas, I.J., et al.	http://signalink.org/
Reactome	Gillespie, M., Jassal, B., Stephan, R., Milacic, M., Rothfels, K., Senff-Ribeiro, A., Griss, J., Sevilla, C., Matthews, L., Gong, C., et al.	https://reactome.org/
HPRD	Keshava Prasad, T.S., Goel, R., Kandasamy, K., Keerthikumar, S., Kumar, S., Mathivanan, S., Telikicherla, D., Raju, R., Shafreen, B., Venugopal, A., et al.	https://www.hprd.org/
DIP	Salwinski, L., Miller, C.S., Smith, A.J., Pettit, F.K., Bowie, J.U., and Eisenberg, D.	https://dip.doe-mbi.ucla.edu/dip/Main.cgi
IntAct	Orchard, S., Ammari, M., Aranda, B., Breuza, L., Briganti, L., Broackes-Carter, F., Campbell, N.H., Chavali, G., Chen, C., del-Toro, N.,	https://www.ebi.ac.uk/intact/home
BioGrid	Stark, C., Breitkreutz, B.-J., Reguly, T., Boucher, L., Breitkreutz, A., and Tyers, M.	https://thebiogrid.org/
ZINC15	Irwin, J.J., and Shoichet, B.K.	https://zinc15.docking.org/
KEGG	Kanehisa, M., Furumichi, M., Sato, Y., Ishiguro-Watanabe, M., and Tanabe, M.	https://www.genome.jp/kegg/

### Resource availability

#### Lead contact

Further information and requests for resources and reagents should be directed to and will be fulfilled by the lead contact, Tero Aittokallio (tero.aittokallio@helsinki.fi).

#### Materials availability

This study did not generate new unique reagents.

### Experimental model and study participant details

Glioma stem cells obtained from the Glioma Cellular Genetics Resource (GCGR, Edinburgh, gcgr.org.uk) are unique, patient derived cell lines – hence no reference sequence is available after STR typing. Classification of these cell lines was performed at the DKFZ (German Cancer Research Centre in the Helmholtz Association), Heidelberg University Hospital (see Table S2).

#### Tissue culture/cell maintenance

*Materials Required* (see Table S3).

##### Cell maintenance-thawing cells

When ready the vial should be quickly thawed in a 37°C water bath. The thawed aliquot should be dispered into 10ml of cold wash media and centrifuge for 3 minutes at 1.5K rpm. One should aspirate as much supernatant as possible and re-suspend in complete media (8ml, containing EGF and FGF) and laminin (10ug/ml) and transfer to a T25 flask. Then, incubate overnight at 37°C and 5% CO_2_. Once the cells have adhered to the plastic, fresh complete media, containing EGF, FGF, and laminin (10ug/ml for E57, 4ug/ml for other cell lines) is added. Take note that it may take a few days to recover. Do not allow to become approximately >80% confluent. Usually the flask will need to be split into a T75 after 48 hours post thaw. Lastly, do not split more than 1:5. The usual ratio is 1:2 or 1:3.

##### Cell maintenance - Passaging cells

For the cell passaging procedure, the media should be removed and washed with 15ml PBS. This is followed by aspirating PBS and adding 1ml accutase in order to coat the bottom of each flask. To ensure complete coverage the flask should be rocked. Then, incubation follows for 3-4 mins at 37°C and 5% CO_2_. Then, 9ml of wash media is added, and the contents are transferred to 15ml conical tube, appropriate for centrifuge (for 3 mins at 1.5 rpm). The supernatant is then aspirated and the pellet is resuspended in 10ml complete media, EGF, FGF and Laminin (4ug/mL) in a T75 flask. The final volume of media is 15ml and 45ug laminin (150ug laminin for E57 cells), but the split can be done as preferred. We note that one should not split more than 1:6 (usually 1:3 a few days before plating) and not allow the split to become (approximately) >80% confluent. Yield is approximately 3x0^6^ cells per T75 flask and viability is usually 80-90%. We also note that doubling time is approximately 24 (E57) to 60 (other lines) hours. Cells tend to form spheroids when becoming too confluent and indicates they should be split. Spheroids can be recovered and will re-adhere. Also, even if cells do not need to be passaged, fresh media should be refreshed after 4 days to encourage healthy growth. Lastly, cells grow faster with prolonged culture. For this reason, passage should exceed 30 and the lowest passage should be used for screening.

##### Cell maintenance - Freezing cells

The freezing media consists of wash media and 10% DMSO. Cells should be detached as described above, counted and resuspended in freezing media at approximately 1-5-2x10^6^ cells per ml, and 1ml for thawing into a T25 flask. Then the cells should be transferred to a specialised freezing chamber and freeze overnight at -80°C, and as soon as possible to liquid nitrogen storage.

#### 384-well plating/dosing

*Materials/Equipment Required* (see Table S4).

##### 384-Well plate pre-coating with laminin

For the pre-coating of the wells, we start first by preparing a diluted solution of laminin (10ug/ml) in complete media. Then a ViaFill dispenser is prepared under sterile conditions, and is followed by adding solution of 20ug/well and incubating at 37°C and 5% CO_2_ for a minimum of 2 hours. Then 25uL of cell suspension (without aspiration) is added at 500-1500 cells/well.

##### 384-Well plate seeding

Cell suspensions should be prepared as described above, followed by pelleting cells and resuspending those in wash media. The cells are then counted. A ViaFill dispenser is prepared under sterile conditions, and the cell suspension is prepared in complete media (without laminin), seeding at 1000 cells/25ul (E3, E21, E31, E34), 500 cells/25ul (E57) and 1500 cells/25ul (E28), and adding 25ul per well, resulting in a final volume of 45ul (∼4ug/ml laminin). Incubation for 30 min at RT is followed, and then plates are transferred to an incubator at 37°C and 5% CO_2_, and are incubated overnight prior to dosing with the library. We noted that for positive control, Staurosporine (1uM) is used for cell death and all wells have final DMSO concentration of 0.1% (w/w).

##### 384-Well plate library dilution and dosing

Library plates were supplied as assay ready microplates (10mM stock in DMSO, 150nL per well, Ebner Group, Oxford). The plates were initially diluted in complete media (50uL/well, 30uM, 1% DMSO (v/v)) using a ViaFill liquid dispenser. The plates were transferred to a BioMek Liquid handling Robot and then serially diluted 1:10 to 3uM, 300nM, 30nM, 1% DMSO (v/v)). Staurosporine (10uM) was added to column 1 of each compound plate. Finally, 5uL of each diluted compound was transferred to the cell plates (1: 10 dilution, final volume 50uL, 0.1% DMSO (v/v) in all wells including ‘untreated controls’, final compound concentrations 3000, 300, 30, 3nM. The plates were incubated at 37°C and 5% CO_2_ for 72 hrs.

#### 384-Well plate fix/stain/acquisition

*Materials/Equipment Required* (see Table S5).

##### Fixation

First the plates are removed from the incubator and are allowed to cool for approximately 10 minutes. A fresh solution of 8% formaldehyde in PBS is prepared. An equivalent volume of fixative is added and incubated at RT for 30 minutes. Then, the entire well is aspirated and washed 3 times with PBS (see Table S6).

##### Permeabilisation

For the permeabilization, the plates are washed 2 times with PBST, incubated in PBST at RT for 20 minutes (minimum) and then aspirated (see Table S7).

##### Hoechst staining

For the staining, the plates are washed 3 times in PBS. Then Hoeschst is added in PBS (1:5000) and then incubated for 30 min in RT in dark. Following that, the plates are washed 3 times with PBS and Hoechst and are sealed and stored at 4°C. Nuclei counts are carried out via built in software module (Molecular Devices), followed by a preliminary univariate analysis using TIBCO Spotfire Analyst, normalized to DMSO wells/plate (see Table S8).

#### Appendix A

For aspirating and dispensing settings, please see Table S9.

### Method details

#### Approved and investigational compound (AIC) collection

We manually selected and curated compounds in the AIC collection based on various sources (Table S10). The compounds’ IDs (from PubChem^58^ and ChEMBL^25^) and their structural description (canonical SMILES) were retrieved using PostgreSQL.^59^ One compound (TAK-530) was found to have neither a compound ID nor a canonical SMILES, and it was therefore removed. The search for approved and investigational compounds for GBM was carried out manually by searching the clinical trials database (https://www.clinicaltrials.gov/), and the availability of these compounds was later determined by crosschecking the compound in SelleckChem (https://www.selleckchem.com/) and PubChem (https://pubchem.ncbi.nlm.nih.gov/). The ECFP4/6 and MACC fingerprint descriptors of compounds were enumerated using the RDKit^60^ chemoinformatics module in Python 3.7.^61^ Dice similarity for ECP4/6 and Tanimoto for MACCS fingerprints with cutoff of ≥ 0.99 was used to identify and remove highly similar compounds^62^ (e.g., doxorubicin and epirubicin, with the first compound being removed). The resulting AIC collection consists of a total of 546 unique compounds, available in GitHub (https://github.com/PaschalisAthan/C3L).

#### Experimental probe compound (EPC) collection

For the EPC collection, compound-target pairs were extracted manually from pan-cancer studies using public databases (PharmacoDB^22^ and The Human Protein Atlas^21^). The wild type and mutant variants of the targets, along with the first neighbors and influencers of other cancer-related targets,^29^ were later used to search for additional compounds that demonstrated sufficient target activity using PostgreSQL^59^ (see the below subsections for details). Next, several compound filtering procedures were applied to generate the three EPC collections (theoretical, large-scale and screening set) using RDKit,^60^ where the filtering procedures involved checking the structural similarity between the compounds, and Python scripts for additional processing, such as adding extra annotations for the targets or the compounds (Figure S3). The full list of cancer-associated protein targets and their probe compounds can be found at https://github.com/PaschalisAthan/C3L.

##### Pan-cancer probe collection (PS1)

This collection of probe compounds focuses on targets and compounds implicated in various types of cancers. To construct this collection, several comprehensive large-scale pan-cancer studies were analyzed (see Table S11). A set of 946 unique targets and 1525 compounds were curated from the pan-cancer studies using the nominal targets from the PharmacoDB database,^22^ and The Human Protein Atlas database.^21^ After removing redundant compounds, a total of 851 unique compounds remained that covered the target space of 946 unique proteins.

##### Extending the compound space (PS2)

To further extend the compound space, the annotated primary targets from the pan-cancer studies were queried across publicly available compound bioactivity repositories.^25^^,^^26^^,^^27^ To include also potential off-targets of the compounds, we used a relatively liberal activity threshold of ≤ 1000 nM for multi-dose bioactivity (IC_50_, EC_50_, K_i_ or K_d_) to identify the compounds having cellular activity against these targets. In case of multiple entries corresponding to the same compound-target interaction, the median bioactivity value was recorded, similar to a previous study.^65^ This curation procedure resulted in 141,087 unique compounds against 441 targets (Table S12).

##### Collection for the mutant target space (PS3)

In addition to investigating the wild type targets implicated in various cancer types, we also identified compounds with cellular activity against their corresponding mutant variants. The mutation information of the annotated targets of Section Pan-cancer probe collection (PS1) were retrieved from the COSMIC Database.^28^ We used various types of variants:

• Substitution - missense
• Substitution - coding silent
• Complex - compound substitution
• Insertion - in frame
• Complex - deletion in frame
• Substitution - nonsense
• Deletion - in frame

We queried these mutant targets across the existing data repositories ChEMBL^25^ and DTC,^26^ and the corresponding compounds were compiled using the criteria similar to those described above. The resulting mutant collection consists of 944 unique compounds targeting 293 unique mutant targets (Table S13).

##### Extending the target space (PS4)

To further extend the target space, we queried public repositories for cancer-related targets, their first neighbors and influencers in four cancer types with high mortality rate (colon, breast, hepatocellular, and non-small cell lung cancer). Such extended “targets” are suggested to influence cancer pathogenesis and therefore to increase the drug target space for anticancer therapies.^29^ Here, a target was considered as a cancer-related gene, when the corresponding protein was either mutated or had a differential expression in cancer. A first neighbour of cancer-related protein was defined as a protein that is directly and physically interacting with a cancer-related protein in human interactome or signalling networks,^29^ according to the databases SignaLink v2,^66^ Reactome,^67^ HPRD,^68^ DIP,^69^ IntAct,^70^ BioGrid,^71^ or in a cancer signalling network.^72^ An influencer protein was defined as a protein that has a direct interaction to one of the first neighbours. After combining these three subsets, an overlap analysis with the cancer-related targets was performed to identify a total of 1115 unique targets. The non-overlapping targets were queried in ChEMBL^25^ and DTC^26^ to find a total of 208 653 potent compounds following the same procedure as in the previous sections (Table S14).

#### Reducing the number of compounds

The full theoretical EPC probe set consists of a total of 336 758 compounds against 1655 unique protein targets (Figure 1). We next reduced the number of compounds to a more manageable size and constructed a compound library that is more attractive to academic screening projects using several filtering procedures, each with freely adjustable cut-off parameters that determine the stringency of the compound filtering process. Figure S4 summarizes the filtering steps and the specific filtering parameters we set in this study to produce the large-scale probe set.

##### Target-specific activity filtering

The first compound filtering technique was to apply a target-specific activity threshold to reduce the number of compounds from the theoretical set with the following steps:

1. All the activity values were log_10_-transformed.
2. Repeat for each target:
  2.1. Target’s bioactivity distribution (IC_50_, EC_50_, K_i_ or K_d_ readouts) was normalized to zero mean and unit variance (z-score normalization).
  2.2. A threshold was selected such that 80% of the target’s activities (80% percentile) remain within that threshold (see an example in Table S15).
  2.3. Compounds with activities higher than the selected threshold were removed (i.e. these show less potency toward the particular target).

##### Compound structural similarity filtering

The next compound filtering step was to find the similarity threshold above which two compounds were considered sufficiently similar. This procedure was based on the assumption that similar compounds are expected to have similar activity distributions. The cut-off value for the Tanimoto similarity was identified with the Akaike Information Criterion, which is an estimator for the degree of information that is lost when using a candidate model, where smaller values indicate low information loss.^73^ More specifically, the following procedure was used for defining the similarity cut-off:

1. A portion (here, 10%) of the total number of compounds was chosen for the similarity cut-off estimation to reduce compound space and computation time.
2. Each compound’s Dice similarity for ECFP4/6 and Tanimoto coefficient for MACCS fingerprints was computed with the rest of the selected compounds.
3. A coefficient threshold was varied from 0.1 to 0.99 (with 0.01 step size), and for each threshold:
  3.1. Compounds that had similarity value equal or greater than the particular threshold were identified.
  3.2. The similarity distributions of those compounds were compared using the Kolmogorov-Smirnov (K-S) test.
  3.3. The Akaike Information Criterion (AIC) value was calculated based on the K-S test statistic values.
4. The optimal similarity threshold was selected based on the smallest AIC value (see Figure S5 for an example).
5. Steps 1-4 were repeated three times using three different random seeds.

##### Global activity filtering

The first step when reducing the size of the physical screening library was to apply an activity threshold similar to that described above, but instead of being target-specific, here the same activity threshold was used across all the targets (i.e., target-agnostic, global filtering). Since two of the theoretical sets are already small in size (PS1 and PS3), the global activity filtering was implemented on the two larger subsets only (PS2 and PS4). More specifically, the procedure followed the steps:

1. The profile of quantitative bioactivity values for each target were recorded when creating the theoretical probe set (see Section 2.1 for details).
2. The bioactivity values were log10-transformed and normalized to zero mean.
3. The target with the highest activity standard deviation was identified.
4. An activity threshold was selected such that 95% of the bioactivities of the targets from step 3 are within the selected threshold (see Figure S6).
5. The selected activity threshold was applied to all the targets to remove the compounds with bioactivities larger than the selected threshold.

##### Reducing the compound space

The second step in the physical screening library size reduction was to pick only a single compound for each target, with the aim to come up with the smallest number of most potent compounds that cover the same target space. More specifically, the compound that had the lowest bioactivity value among the multi-dose activity types (IC_50_, EC_50_, K_i_, K_d_) was selected for the particular target, since that compound is assumed to have the highest binding potency against the target. The different activity types were treated equally in this process due to sparsity of bioactivity readout data.

##### Compound availability filtering

The final step for designing the physical screening library was to include only those compounds that are available for sale from at least one vendor using the information from ZINC15.^74^ More specifically, if a particular compound was not available for sale, at the time of library curation, then the next most potent compound that is available for sale replaced the original one in the screening library. Even though this step reduced the number of compounds in the final screening library by 52% of the original size, the targets’ coverage remained at 86% of the original target space (see Table S16). Furthermore, the target activity distributions remained relatively unchanged (Figure S1), and the differences originated mainly from the few of the most potent compounds.

#### Cell survival profiling of glioblastoma patient cells

We performed high-throughput screening of the GBM patient glioma stem cells with an image based, nuclei staining technique. In this pilot application of the C3L design principles, the cell-based assay was performed using 789 compounds in the physical library, which included 325 of the screening set compounds (27%). The rest of the screening set compounds were not timely available at the time of library curation, or they were prohibitively expensive to purchase from any vendor. To compensate for this, we sourced additional 464 theoretical set compounds, which were selected to maximize the target space. In total, the pilot physical library covered 1320 out of the 1655 targets (80%) of the screening set. In this pilot screening study, we sourced the compounds first through collaborators, and then from commercial providers, by focusing on the most potent compounds against as many of the 1655 protein targets for comprehensive cell-based testing.

##### Patient samples and cell-based assay

The 789 compounds were tested on 6 patient-derived GBM stem cell models from Glioma Cellular Genetics Resource (http://gcgr.org.uk), with 2 patients from each molecular subtype of GBM that are characterized by differences in gene expression and genetic alterations:^33^ classical (n=2), proneural (n=2), and mesenchymal (n=2). The protocol comprises of the following steps^13^ (see Figure 4A):

1. Cells were seeded (500-1500 cells/well) onto pre-coated laminin (10 ug/mL) 384-well plates (Greiner Bio-One microclear plates, 781091) using an INTEGRA VIAFILL dispenser. Final laminin concentration was 4ug/mL.
2. The cells were incubated overnight at 37°C, 5% CO_2_.
3. Assay ready compound plates were serially diluted in media using a Biomek liquid handling robot (Beckman). The final concentrations used included 3-30-300-3000nM with DMSO (0.1% w/w) as negative control and staurosporine (1uM) as positive control (for cell death). All wells had a final DMSO concentration of 0.1% (w/w).
4. The cells were incubated for 72 hours.
5. Cells were fixed *in situ* with formaldehyde and then permeabilized with TX-100 (0.01% w/w in PBS. Between steps, plates were washed using a BioTek plate washer (Agilent).
6. Nuclei staining (Hoechst 33342, 2ug/mL in PBS, 20 minutes, room temp).
7. Plates were washed and sealed with aluminium microplate seals (StarSeal, Starlab Group).
8. Image acquisition using ImageXpress Micro Confocal High-Content Imaging System (Molecular Devices).
9. Image analysis and quantification using MetaXpress Software (Molecular Devices, v. 6.7.2.290), analysis module 'Count Nuclei'.
10. Data processing and univariate analysis using TIBCO Spotfire Analyst (PerkinElmer, version 10.10.3.17), normalized to DMSO wells.

##### Survival fraction, *Z* score and AUC calculation

While there are multiple imaging features, such as shape and granularity, that can be quantified for each cellular compartment (nuclei, cytoplasm, etc), here we used only one feature for further analysis: the survival fraction and its z-score (see Figure 4A) from staining the nucleus with Hoechst 33342. The survival fraction is calculated by dividing the nuclei count of the treated sample by the nuclei count of the untreated control sample (DMSO 0.1% (v/v)), and the z-score is the number of standard deviations from the mean survival fraction normalized to DMSO. The lower the survival fraction or the z-score the more effective is the compound in the sense that fewer cancer cells survived the treatment or that the survival score is lower than the mean survival score of the negative controls, respectively. To summarize the activity of a compound across the 4 tested concentrations, we calculated the area under the dose-response curve (AUC) using trapezoidal rule in NumPy^75^ for each compound-patient pair using the survival fraction as the response readout (see Figure 4C).

##### Data-driven activity threshold definition

To set an activity threshold for strong hits based on the nuclei count measurements, we applied a mixture modelling^76^ on the survival fraction distributions, separately for each concentration and patient (but combining all the GBM subtypes due to the small number of samples in each subtype, n=2). As expected for molecularly-targeted compounds, most of the responses appear around 1 in terms of survival fraction (see Figure 4B). To identify extreme compound responses falling outside of these background distributions, we estimated a mixture model with two Gaussian components, one component for the background responses (centered around 1 after parameter estimation), and the other distribution for the extreme responses (parameters freely estimated from the patient-specific survival fraction data). To identify compound responses far enough from the background distribution, we chose a cut-off of 0.01 percentile of the background distribution as the activity threshold to classify a compound as a strong hit (green dotted lines in Figure 4B). In comparison to a fixed threshold based on SF levels, or their z-scores, such data-driven activity threshold ensures that the hit selection does not only depend on the full distribution of the measurements, but the activity threshold is also patient- and concentration-specific, and therefore takes into account potential cell growth and dose-response differences. Finally, the compounds with responses under the activity thresholds across all the 4 concentrations were classified as strong hits for further analyses.

##### Pathway analyses and web-based data platform

The target annotations available in C3L were used to map the strong hit compounds to molecular pathways and cellular processes. We used Reactome pathway tool^67^ and KEGG pathways^77^ (through ShinyGO tool^78^) to explore the pathways that are modulated by the strong hits using the pre-selected activity cut-off of 0.01 percentile of the background distribution (see the section Data-driven activity threshold definition above). We have also implemented an interactive web-application (www.c3lexplorer.com) that enables users to freely set various compound selection criteria, including data-driven activity threshold for the active compound selection, and then interactively explore drug-target interactions and pathways targeted by the selected compounds for additional analyses and hypothesis generation.

### Quantification and statistical analysis

In the section Compound availability filtering*,* Kolmogorov-Smirnov test was used in Python to test whether there was any difference on the distribution of the compound-target activities before and after replacing the unavailable compounds in the screening set. A significance value of p<0.05 was considered to indicate a statistically significant difference.

### Additional resources

Data exploration and visualization web-platform: http://www.c3lexplorer.com/.

## Data Availability

• All data has been deposited at Zenodo (https://zenodo.org/record/7945379), and assay annotations are available at GitHub (https://github.com/PaschalisAthan/C3L). • This paper does not report original code. • Data exploration and visualization web-platform: http://www.c3lexplorer.com/.
