# Supplementary material for: Chemogenomic library design strategies for precision oncology, applied to phenotypic profiling of glioblastoma patient cells
Source: iScience. 2023 Jun 25;26(7):107209. doi: 10.1016/j.isci.2023.107209 (PMC10359939; doi:10.1016/j.isci.2023.107209)
Supplement: Document S1. Figures S1–S7 and Tables S1–S16 [file mmc1.pdf]

iScience, Volume *26*

Supplemental information

Chemogenomic library design strategies for precision oncology, applied to phenotypic profiling of glioblastoma patient cells

Paschalis Athanasiadis, Balaguru Ravikumar, Richard J.R. Elliott, John C. Dawson, Neil O. Carragher, Paul A. Clemons, Timothy Johanssen, Daniel Ebner, and Tero Aittokallio

Supplemental Figures

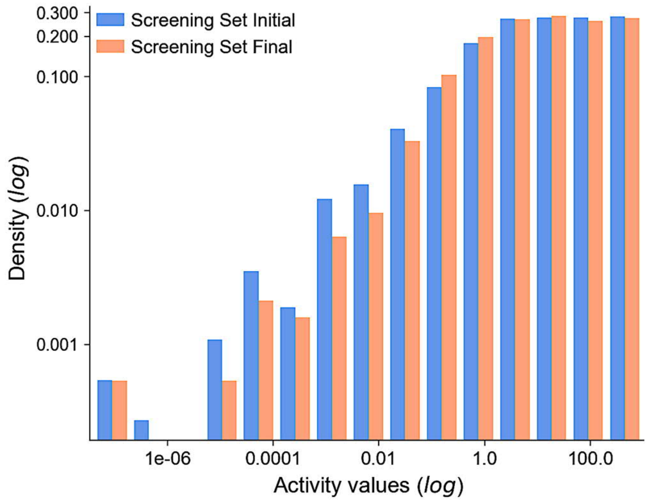

Figure S1. Comparison of the compound-target activities before and after replacing the unavailable compounds in the screening set, related to Figure 1. The x-axis and y-axis are log_{10}-scaled, while the numbers present the non-logged values. The activity distributions were generally similar ($p>0.05$; Kolmogorov-Smirnov test).

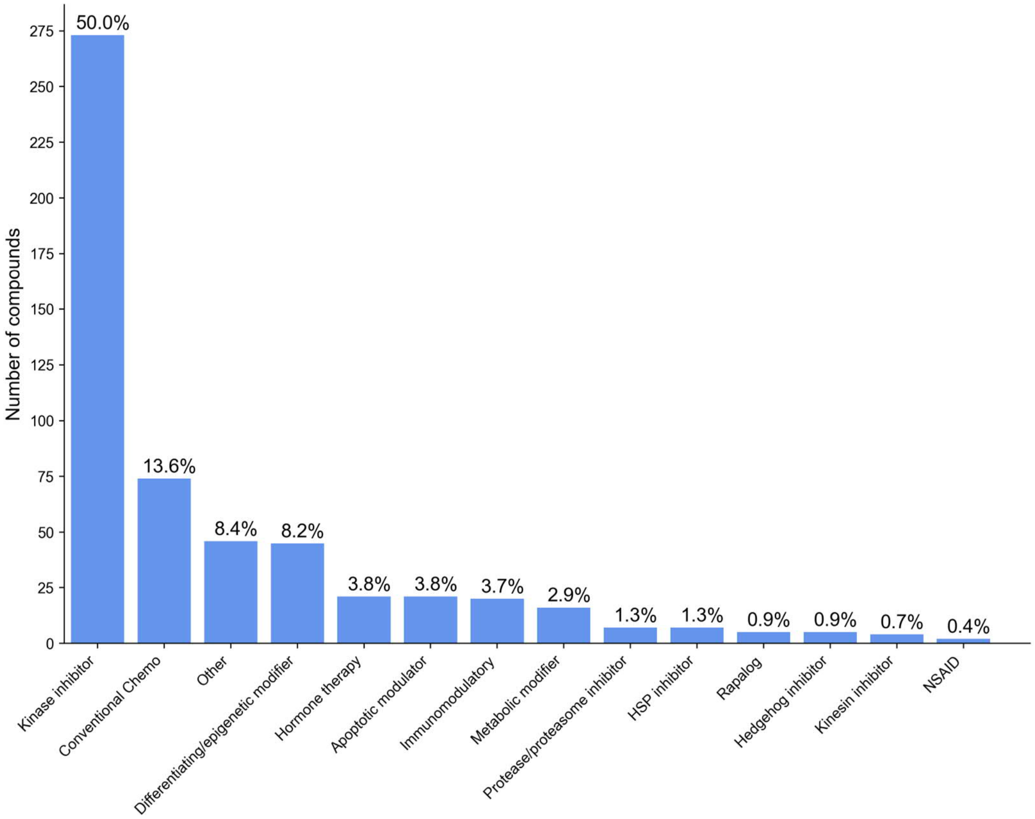

Figure S2. The number of AIC compounds across drug classes, related to Figure 2. Each compound was categorized based on its broader target/mechanism class using information from FIMM, DrugBank and SelleckChem. HSP, Heat Shock Protein; NSAID, Non-Steroidal Anti-Inflammatory Drugs. The numbers above the bars indicate the percentage of compounds in the drug classes.

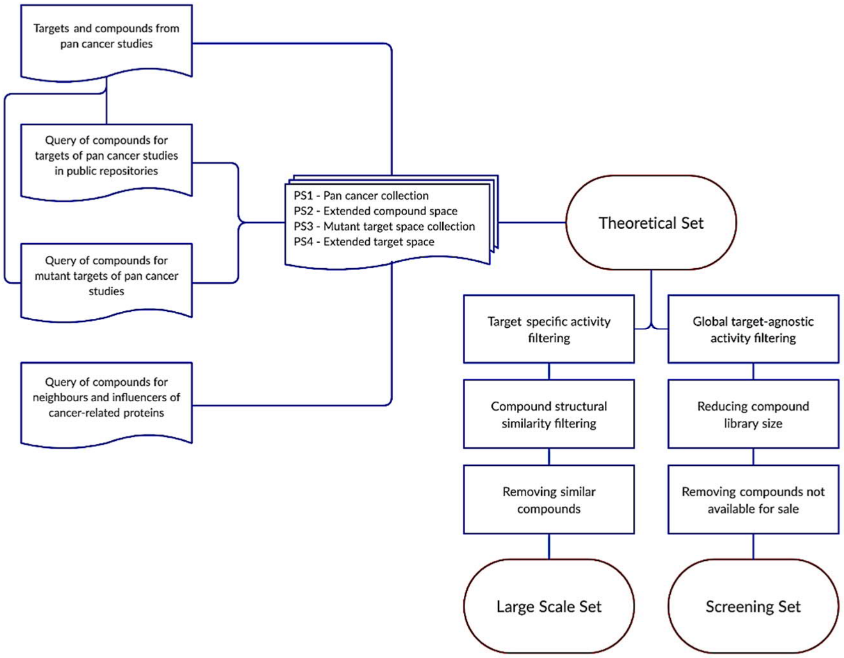

Figure S3. Workflow of generating the three probe compounds sets (Theoretical, Large-scale and Screening set), related to STAR Methods. Four probe sets (PS) were defined: the pan-cancer collection (PS1) includes compounds and their annotated nominal targets from various pan-cancer studies. The extended compound space (PS2) consists of compounds that have off-target activity against the annotated targets of the pan-cancer studies, while excluding compounds already in the PS1 set, retrieved from public drug/target repositories such as ChEMBL[25], Drug Target Commons (DTC)[26] and DrugBank[27]. The collection of the mutant target space (PS3) consists of compounds that have activity against the mutant variants of the annotated targets extracted by using the COSMIC database[28]. The extended target space collection (PS4) extends the target space of cancer-related targets through the nearest neighbor approach[29]. See text for the further details of the workflow.

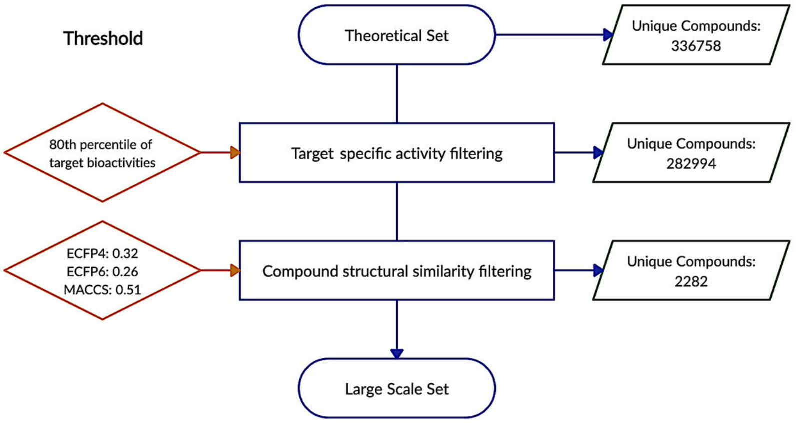

Figure S4. The design procedure for the large-scale probe compound set, related to STAR Methods. The target-specific activity filtering is based on either biochemical or cell-based evidence that the compound has potency against the particular target (less stringent filtering). Compounds that show sufficient similarity to other compounds based on two out of three fingerprints (ECFP4/6 and MACCS) were removed in the structural similarity filtering step (more stringent filtering). The thresholds used in the current design procedures are shown on the left.

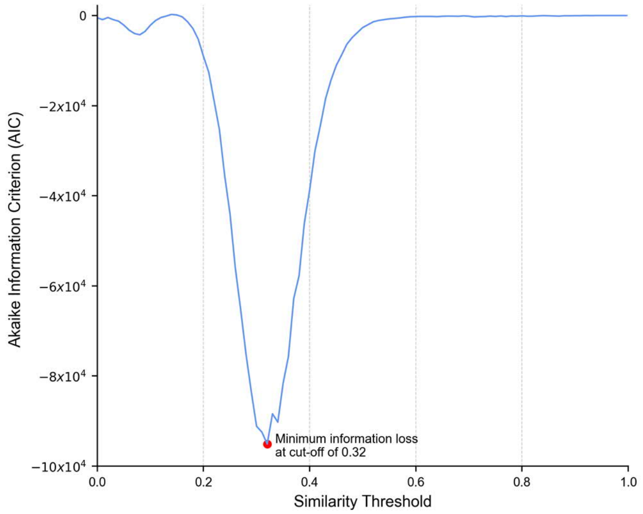

Figure S5. An example how optimal similarity threshold was identified based on Akaike Information Criterion, related to STAR Methods. The optimal structural similarity threshold was defined for each structural fingerprint separately (ECFP4, ECFP6 and MACCS), as the smallest value of the curve (thresholds ranging from 0 to 1), indicating the lowest information loss.

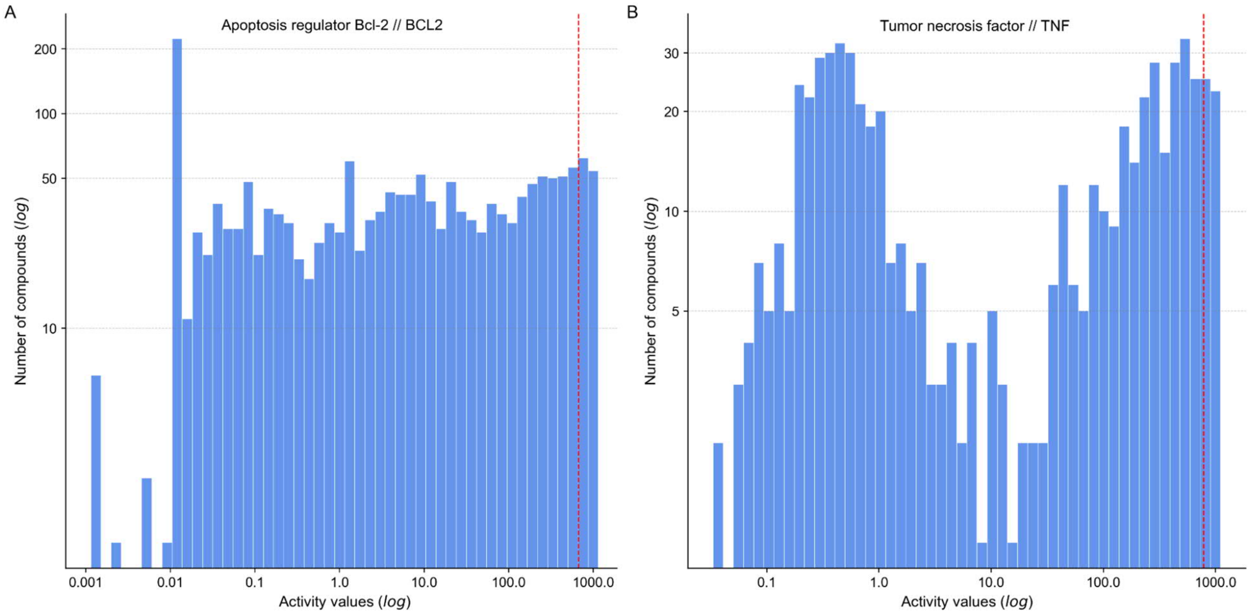

Figure S6. Examples of protein targets with highest standard deviation of compound bioactivities for identifying the global activity threshold, related to STAR Methods. (A) Activity threshold of 670nM (red dotted line) in the PS2, and (B) activity threshold of 780nM (red dotted line) in the PS4.

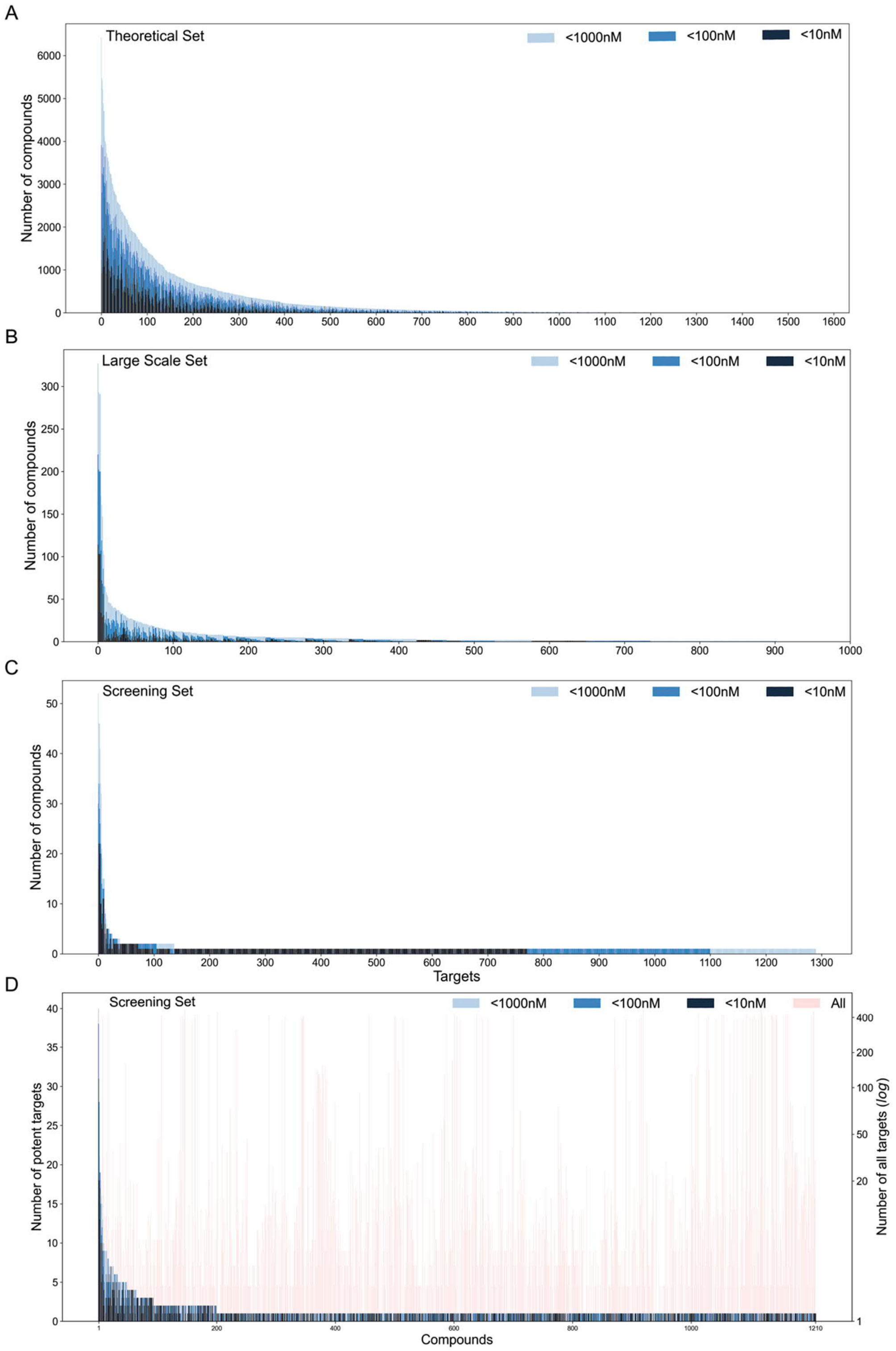

Figure S7. Number of compounds against targets using three activity thresholds, related to Figure 3. (A-C) Compounds in the three probe collections with different library sizes. Note the differences in the y-axis ranges. (D) Number of potent targets for the compounds in the Screening set using three activity thresholds. The right-hand y-axis depicts the total number of multi-dose bioactivity data for each compound-target pair available in Drug Target Commons, ChEMBL and DrugBank. The y-axis is $\log_{10}$-scaled while the numbers present the non-logged counts.

Supplemental tables

Table S1. The compound and target spaces of the probe compound collections, related to STAR Methods.

Probe collection	Unique compounds	Unique protein targets	Unique interactions*
Theoretical set	336 758	1655	494 320
Large-scale set	2285	1655	8137
Screening set	1211	1386	2699

Unique compound-target activities were calculated by considering various bioactivity types from multi-dose assays as repeated measurements of the same compound-target interaction

Table S2. Patient-derived glioma stem cell lines, related to STAR Methods.

GCGR ID	Sample Identifier	Sentrix ID	Array Type	Gender	Class	Subtype*
E13	sampleName1527692409	202010290060_R02C01	EPIC	Female	GBM, IDH^{wt}	Classical
E21	sampleName1527692116	202047860190_R07C01	EPIC	Male	GBM, IDH^{wt}	Mesenchymal
E28	sampleName1527692835	202010290118_R08C01	EPIC	Female	GBM, IDH^{wt}	Classical
E31	sampleName1540663815	202262730090_R02C01	EPIC	Male	GBM, IDH^{wt}	Proneural
E34	sampleName1540663855	202262730090_R04C01	EPIC	Male	GBM, IDH^{wt}	Proneural
E57	sampleName1573854087	203057570091_R05C01	EPIC	Female	GBM, IDH^{wt}	Mesenchymal

* Subtype classification also based on RNA-Seq data

Table S3. Materials required for tissue culture and cell maintenance, related to STAR Methods.

Description	Item	Supplier	Catalogue no.	Amount per 500mL
Complete Media	DMEM/HAMS-F12 (500mL)	Sigma	D8437-6x500mL	N/A
Cell Maintenance	Penicillin-Streptomycin (10K U/mL, 100mL)	GIBCO/Life tech	15140-122	5mL

Media				
	BSA solution 7.5% (100mL)	GIBCO/Life tech	15260-037	0.8mL
	Glucose (100g/L, 100mL)	Sigma	G8644	7.5mL
	beta-mercaptoethanol (50mM, 20mL)	GIBCO/Life tech	31350-010	1mL
	B27 Supplement (50x, 10mL)	GIBCO/Life tech	17504-044	5mL
	N2 Supplement (100X, 5mL)	GIBCO/Life tech	17502-048	2.5mL
	MEM Non-Essential Amino Acids (100X, 100mL)	GIBCO/Life tech	11140-035	5mL
	rhFGF basic (100ug/mL)	PEPROTECH	100-18b	60uL
	rmEGF (100ug/mL)	PEPROTECH	315-09	60uL
	Laminin (1mg/mL)	R&D Systems/Biotechne	3446-005-01	4ug/mL (add fresh)
Wash Media	DMEM/HAMS-F12 (500mL)	Sigma	D8437-6x500mL	N/A
	Penicillin-Streptomycin (10K U/mL, 100mL)	GIBCO/Life tech	15140-122	5mL
	BSA solution 7.5% (100mL)	GIBCO/Life tech	15260-037	0.8mL
Sub-culture	Accutase (100mL)	Sigma	A6964-100ML	N/A

Table S4. Materials required for 384-well plating and dosing, related to STAR Methods.

Description	Item/Notes	Supplier	Catalogue no.
Complete/Wash media	As above		
Seeding Media	As above for complete media, but no EGF or FGF added		
Accutase			
Cell Suspension	G7-SOX2-V5 cells, prepared as above (re-suspended in wash buffer prior to cell count)		
Plates	384 Well, black, transparent, sterile	Greiner Bio-one	781091 (or equivalent)
Laminin (10ug/mL)	Prepared from stock listed above in PBS		
PBS			
Integra ViaFill	Microplate liquid dispenser		
Cell Counter/Trypan Blue			
BioMek (Beckman)	96 Head Liquid handling robot and p20 tips (sterile)		

Table S5. Materials and equipment required required for 384-well plate fixation, staining and acquisition, related to STAR Methods.

Description	Item/Notes	Minimum Volume per plate	Supplier	Catalogue no.
Fixative	8% formaldehyde solution in PBS	25mL (50uL/w)	From 37% stock, Fisher Scientific	10532955
Wash Buffer #1	PBS	100mL (50uL/w)		
Permeabilisation	PBST - TX-100 0.1% (v/v)	25mL		

Buffer		(50uL/w)		
DAPI or Hoechst stain	25mL per plate (1:10000 or 1:5000) in PBS	25mL (50uL/w)	Hoechst-33342, MolProbes	H1399
Multidrop	Microplate liquid dispenser			
Plate washer				
Imagexpress	High content microscope		Molecular Devices	

Table S6. Fixation steps, related to STAR Methods.

Step	Action	Vol./well	Suggested Equipment/Notes
1	Remove plates from incubator and allow to cool (approx. 10 min.)		
2	Prepare a fresh solution of 8% formaldehyde in PBS		
3	Add equivalent volume of fixative	50uL	Multidrop Combi. Final conc. 4% formaldehyde
4	Incubate at RT for 30 minutes		
5	Aspirate entire well and wash 3x PBS	50uL x 3	Plate Washer

Table S7. Permeabilisation steps, related to STAR Methods.

Step	Action	Vol./well	Suggested Equipment/Notes
6	Wash 2x PBST	50uL x 2	Plate washer. Retain final wash
7	Incubate in PBST at RT for 20 minutes (minimum)		
8	Aspirate		Plate washer

Table S8. Hoechst Staining steps, related to STAR Methods.

Step	Action	Vol./well	Suggested Equipment/Notes
9	Wash PBS x3	50uL	Plate washer
10	Hoechst in PBS (1:5000)	20uL	Plate washer & liquid handler
11	Incubate 30 min, RT in dark		
12	Wash PBS x3	50uL	Plate washer
13	Seal plate and acquire/store 4 ^{o}C		ImageXpress: DAPI channel, 20X objective, 6 sites per well
14	Nuclei Counts carried out via built in software module (Molecular Devices)		
15	Preliminary univariate analysis using TIBCO Spotfire Analyst, normalised to DMSO wells/per plate.		

Table S9. Aspirating and dispensing settings, related to STAR Methods.

Plate Washer Settings (Biotek-405)	Aspirate Settings (Washing)	TOP
		Travel rate 6 (14.7mm/sec)
		Delay 0000
		X position 00

		Y position 00
		Height 028 (3.557mm)
		Secondary Asp NO
	Aspirate only	As above but:
	(ie before antibody addition)	Height 024 (3.049mm)
		Secondary Asp YES
		Secondary Asp Height 023 (2.922mm)
	Dispense (Washing)	Dispense rate 03
		Vol 80uL
		delay volume 00
		X position 00
		Y position 00
		Height 120 (15.245mm)

Table S10. Sources of the AIC collection, related to related to STAR Methods.

Resource	Number of compounds	Source database
Approved kinase inhibitors	62	FDA-approved kinase inhibitors retrieved manually from a review article[a]
FIMM oncology collection[b]	460	Manual curation of small-molecules for their approval status in different countries
Glioblastoma therapeutics	25	Approved and investigational compounds for GBM from Clinical Trials database[c]

[a] Roskoski, R., Jr (2020). Properties of FDA-approved small molecule protein kinase inhibitors: A 2020 update. Pharmacol. Res. 152, 104609. 10.1016/j.phrs.2019.104609.
[b] FIMM, Institute for Molecular Medicine Finland
[c] https://www.clinicaltrials.gov/

Table S11. Number of compounds targeting intended targets in pan-cancer studies, related to STAR Methods.

Dataset		Number of compounds	References
PharmacoDB	GDSC	404	40
	CTRPv2	342	41,42,43

	CCLE	24	44
	GRAY	74	45
	FIMM	112	46
The Human Protein Atlas		569	21
All compounds		1525	
Unique compounds		851	
Unique targets		946	

Table S12. Number of compounds with activity against the pan-cancer targets, related to STAR Methods.

Repository	Number of compounds	Reference
ChEMBL	140 626	25
DTC	99 565	26
DrugBank	845	27
All compounds	241 036	
Unique compounds	141 087	

Table S13. Number of compounds with activity against the mutant protein variants, related to STAR Methods.

Repository	Number of compounds	Reference
ChEMBL	944	25
DTC	446	26
Unique compounds	944	

Table S14. Number of compounds with activity against the extended target space, related to STAR Methods.

Repository	Number of compounds	Reference
ChEMBL	206 944	25
DTC	178 250	26
Unique compounds	208 652	

Table S15. Activity threshold selection example, related to STAR Methods.

Target	Compound	Activity	Value(nM)*
O14578	CHEMBL379218	Kd	13
O14578	CHEMBL574738	Kd	52
O14578	CHEMBL603469	Kd	85
O14578	CHEMBL558752	Kd	87
O14578	CHEMBL1721885	Kd	94
O14578	CHEMBL428690	Kd	110
O14578	CHEMBL1287853	Kd	140
O14578	CHEMBL494089	Kd	200
O14578	CHEMBL572881	Kd	300
O14578	CHEMBL607707	Kd	310
O14578	CHEMBL388978	Kd	240
O14578	CHEMBL10	Kd	420
O14578	CHEMBL475251	Kd	440 (80th percentile)
O14578	CHEMBL278041	Kd	510
O14578	CHEMBL1908397	Kd	650
O14578	CHEMBL553	Kd	680

*Cells in red are the activities that are retained after applying the target-specific activity filtering. The bioactivities are arranged from most potent (small K_d) to less-potent (high K_d).

Table S16. Compound and targets spaces before and after replacing the unavailable compounds, related to STAR Methods.

Probe set	Unique compounds	Unique compounds for sale*	Unique targets	Unique interactions
Screening set initial	2331	635	1628	5096
Screening set after compounds replaced	1211	1211	1386	2533
C^3L physical library	789	789	1320	6460

* The commercial availability information was extracted from ZINC15[74].
